# Numerical investigation of injection characteristics on normal saline irrigation quality in curved root canals using computational fluid dynamics

**DOI:** 10.1016/j.heliyon.2023.e23773

**Published:** 2023-12-15

**Authors:** Arash Izadi, Mohsen Lashkarbolok, Ezatolah Kazeminejad, Iman Tavakolinejad Kermani, Yaser Mesri

**Affiliations:** a Dental Research Center, Golestan University of Medical Sciences, Gorgan, Iran; b Department of Endodontics, Faculty of Dentistry, Golestan University of Medical Sciences, Gorgan, Iran; c Department of Civil Engineering, Faculty of Engineering, Golestan University, Aliabad Katoul, Iran; d Research Center for Biomedical Engineering, Ferdowsi University of Mashhad, Mashhad, Iran; e Faculty of Dentistry, Golestan University of Medical Sciences, Gorgan, Iran

**Keywords:** Fluid dynamic, Curved root canal, Injection characteristics, Irrigation quality

## Abstract

**Objectives:**

The primary aim of this research was to find the effects of Normal saline injection rate and position on irrigation quality in curved root canals.

**Methods:**

Computational Fluid Dynamics (CFD) was used to simulate irrigation in root canals. Root canal geometries were obtained by considering the complete shaping of a rotary file in four root groups with 0, 10, 30, and 60° of curvatures. Fluid surface tension, bouncy, gravity, and air entrance were considered in the numerical simulation using Ansys-CFX software. For each root canal, the flow regime and wall shear stresses were calculated at three different syringe plunger speeds, and three different injection locations of a beveled open-ended needle and their effects on the irrigation quality were investigated in this paper.

**Results:**

Wall shear stress is considered the main parameter determining irrigation quality. It was calculated for all 36 test cases. Injection rate and normal saline accumulating near the apex and their effects on washed area ratio were shown for the test cases. Results showed significant effects of injection characteristics, incredibly faster injection, and deeper needle position on the irrigation quality of curved root canals.

**Conclusions:**

A higher injection rate and deeper needle position are required to achieve the best irrigation quality and a more washed area ratio in root canals with greater curvature.

## Highlights

• Computational fluid dynamics have a great potential to analyze irrigant flow behaviors in endodontics.
• The irrigant velocity, position, geometry of the irrigation needle, root canal geometry, and curvatures influence flow dynamics.
• Increasing the curvature from a straight root canal up to 30° canals decreases wall shear stress slightly.
• Interestingly, the average shear stress in 60° root canals increases dramatically. Because of the formation of recirculation zones in highly curved canals.

## 1 Introduction

Debridement is an essential procedure in root canal treatment. Irrigation is the final part of debridement in which the residual debris remaining from canal preparation is removed using an irrigant flow. Normal saline injection into a prepared root canal using a plunger and needle is a common irrigation method. Injection rate, needle location in a root canal, root canal morphology, and irrigant fluid properties affect irrigation quality. Many studies in the endodontic field were carried out experimentally, Ex-Vivo [[1], [2], [3], [4], [5]]. Experimental researches are expensive and may require complicated techniques in variable measurements.

In contrast, numerical simulations are in-expensive and flexible tools that have successfully been carried out in many practical scientific fields. Using numerical packages, or Computational Fluid Dynamics (CFD) tools, in endodontics are relatively new. Boutsioukis et al. [6] investigated the flow patterns created by various irrigant flow rates within a prepared root canal by simulating a syringe and needle using FLUENT 6.2 software. They advised that irrigation needles should be placed within one mm of working length to ensure fluid exchange [6]. Their method was one of the initial attempts to apply a numerical tool to investigate an endodontic problem. Boutsioukis et al. [7] compare the irrigant flow's CFD results within a prepared root canal with experimental visualizations using Particle Image Velocimetry (PIV) and theoretical calculations. Their results showed a good agreement between numerical and experimental results. They also studied the effect of positioning the needle in an off-center location inside the root canal. They concluded that small laterally moving needles inside the canal do not significantly affect the flow [7]. In another study, Boutsioukis et al. [8] investigated the effect of root canal taper on irrigant flow inside a prepared root canal. Their results showed that an increase in the root canal taper results in higher wall shear stress and irrigant replacement and reduces the risk for irrigant extrusion [8]. The impression on flow characteristics such as apical pressure, flow pattern, and flow velocity arises from a variation of irrigant flow rate, depth of insertion, and needle type was studied by Šnjarić et al. [9] using Star- CCM + numerical software. They concluded that these factors influenced irrigant flow patterns [9]. In a comprehensive study, root canal irrigant flow with different irrigation needles was simulated Three-dimensionally using FLUENT by Shen et al. [10]. They found that the type of needle tip impacts flow patterns, velocity, and also apical wall pressure. Hence they recommended CFD tools for irrigant needle tip design. Shirazi et al. [11] simulated the irrigant penetration into dental microtubules using ANSYS Fluent. They concluded that side-vented needles have lower mass transfer ability than open-ended ones at the same flow rate. They also showed that when the flow arrives at the end of the canal near the apex, its penetration to the dentinal tubules reduces [11]. Wang et al. [12] examined the impact of the orientation of a side-vented needle on the velocity, flow, and wall stress distribution in a C-shaped root canal using CFD. They also investigated the parameters such as irrigant replacement and apical pressure in the canal and evaluated the real-time replacement of irrigants in the lateral canal [12]. Ghalandari et al. [13] simulated the flow of silver/water nanofluid in the root canal using Ansys CFX and evaluated the impact of the injection height and fluid concentration on irrigation quality. Hu et al. [14] investigated the influence of needle movement on canal irrigation using Flow-3d CFD software. They proposed that moving the needle ups and downs during irrigation avoids periapical extrusion accidents [14]. In one of the more realistic numerical simulations, in which air entrance was considered in Ansys-CFX, Isvoranu and Danaila investigated the irrigation device's irrigation efficiency [15].

The present study simulates the flow of normal saline in straight and three maxillary curved roots at different injection rates and locations using Ansys-CFX (Ver 19.2). To the author's knowledge, this study also represents the first idealized model in air entrance; bouncy effects, gravity, surface tension, and air pocket formation are considered, which allows the study of several geometries of the root canal. Wall shear stress is assumed to be the main deterministic parameter in irrigation quality, and a criterion is defined to evaluate its significance. Injection rate and needle position are investigated, and their effects on washed area ratio are studied. The present study compares these conditions by considering the effects of inlet velocity and root curvature. Furthermore, the numerical model can examine the area of washed root surface with the help of shear stress values.

## 2 Materials and methods

### 2.1 Geometry

**Fig. 1:**
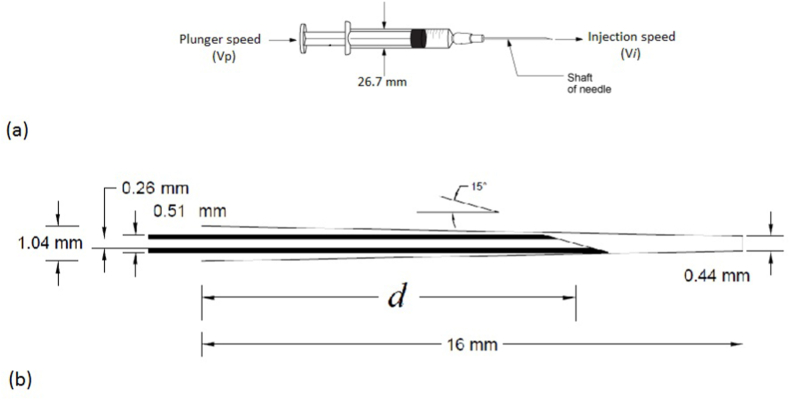
a) Syringe geometry and b) Needle gauge #25 in a straight prepared root canal.

**Figure (2):**
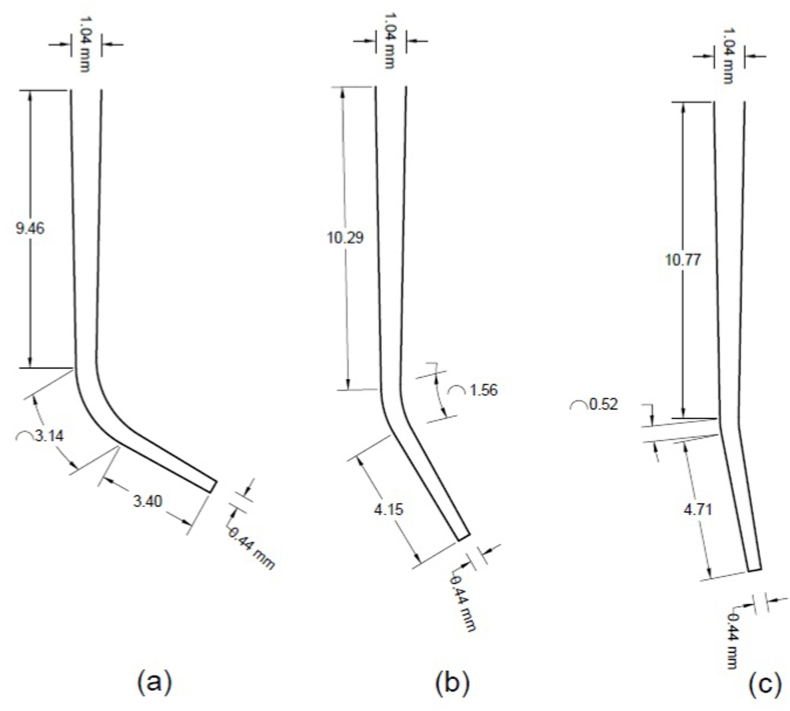
Prepared curved root canal dimensions ((a) 60°, (b) 30°, (c) 10°).

A standard 5 cc syringe is considered a medical device injecting the irrigant fluid into the root canal. A hypodermic needles gauge #25 is used for all simulations. The needle is assumed to inject normal saline while placed at *d* mm (*d* = 4, 6, and 8 mm) from the start point of the root. Straight-prepared root canal geometry and needle size and position are depicted (Fig. 1). The root canal geometries are based on root canal shaping by a rotary-driven Ni–Ti instrument (size 40-4% taper). The root canal curvatures are calculated by the Schneider method (Fig. 2a 60°, Fig. 2b 30° and Fig. 2c 10° curved root canal) [16].

### 2.2 Mesh generation and mesh independency analysis

**Figure (3):**
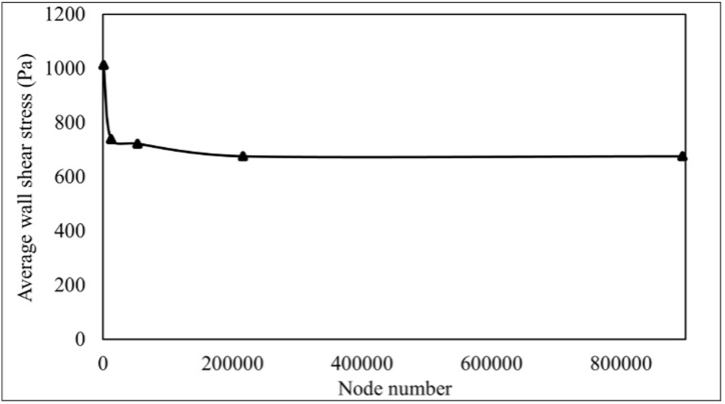
Mesh independency monitoring of solving domain.

Ansys workbench meshing application is used to build the root canal mesh cells considering adaptive size function control. This research performs mesh independency analysis to obtain the optimum mesh size. A coarse mesh is initially constructed with a size of 0.2 mm and 795 nodes. Flow simulations are performed with identical boundary conditions, a 60° curved root canal as geometry, and an inlet velocity of 23.54 m s^−1^. The refinement procedure is performed by evaluating the average wall shear stress of the root canal considering medium (mesh size of 0.05 mm and 12421 nodes), fine (mesh size of 0.025 mm and 53479 nodes), finer (mesh size of 0.0125 mm and 215925 nodes) and finest (mesh size of 0.00625 mm and 895163 nodes) mesh size. The mesh independency procedure is shown in (Fig. 3). The final mesh size of 0.0125 mm comprises 215925 nodes, and 899334 cells are used for all simulation cases.

### 2.3 Boundary and operating conditions

**Figure (4):**
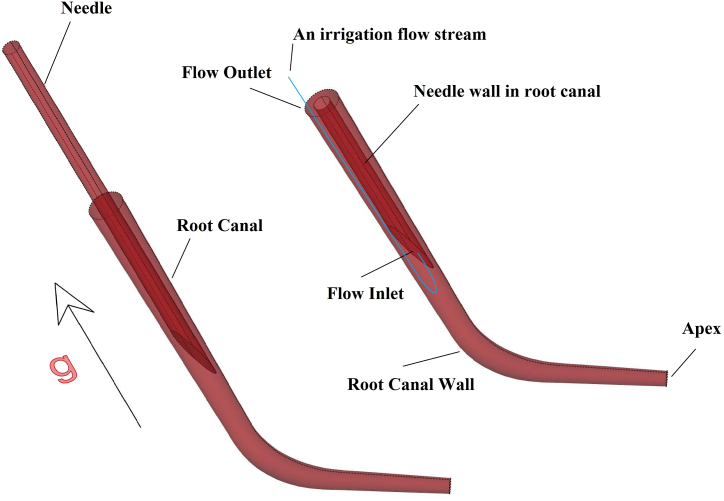
The schematic artwork of root canal boundary.

The irrigation procedure is modeled considering the needle entering the root canal, as shown in (Fig. 4). It is assumed that the root canal is empty of pulp and is full of air at initial condition, then the irrigant flow comes in from the inlet plane of the needle and comes out from the outlet plane on top of the root canal after irrigating the root canal wall. The inlet flow velocity is assumed to be constant. This assumption is an idealized situation to simulate fluid flow compared to realistic conditions. However, injection with variable velocity leads to more realistic results requiring powerful parallel processing devices. Constant inlet velocity helps us to simulate No-slip smooth boundary conditions considering the flow contact with the root canal wall and the needle. Inlet velocity is assumed as a boundary condition of the flow entrance at the needle tip. The plane between the root canal wall and the needle at the top of the root canal is assumed as the outlet plane with atmospheric pressure. Normal saline with a density, ρ, equal to 1.05 (gr/cm^−3^) is used as the irrigant fluid. Gravity acceleration, g, equal to 9.81 m/s^−2^, is assumed in the direction illustrated in (Fig. 4). Inlet velocities equal to 7.84, 11.77, and 23.54 (m s^−1^) are obtained by considering three injection times. Period of pushing the plunger to empty the whole existing normal saline in the syringe, respectively equal to 12, 8, and 4 (s). 36 injection cases are studied according to three different locations of the needle, three injection times, and four roots with 0, 10, 30 and 60° of curvatures (Appendix Table 1).

### 2.4 Initial conditions and solving equations

At the initial condition, the root canal is assumed to be empty. The irrigant flow is considered with a 10 (%) turbulence rate and constant discharge along the y-axis from the needle inlet.

In order to numerically solve the velocity and fluid flow inside the root canal, mass and momentum conservation equations are numerically solved. Details of these equations is represented in Ref. [17].

$$\frac{\partial \rho }{\partial t}+\nabla .\left(\rho \vec{v}\right)=S_{m}$$

where ρ, $\vec{v}$, $S_{m}$ and t stand for fluid density, velocity vector, mass sink/source in the flow domain, and time, respectively.

$$\frac{\partial \left(\rho \vec{v}\right)}{\partial t}+\nabla .\left(\rho \vec{v}.\vec{v}\right)=-\nabla p+\nabla .\bar{\tau }+\rho \vec{g}+\vec{F}$$

where p, $\vec{g}$, $\bar{\tau }$ and $\vec{F}$ refers to pressure field, gravitational acceleration, tensor of tension, and external force.

Furthermore, the applied model for turbulence is k-ε. Details of this model are represented in Ref. [18].

## 3 Results

**Figure (5):**
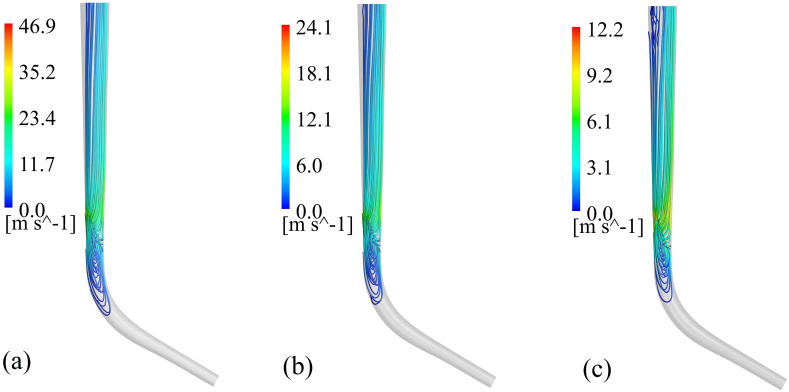
Streamlines of normal saline flow in 60° curved root canal with injection times of (a) 4 (b) 8 and (c) 12 (s).

The irrigation procedure of the curved root canals is studied using 36 numerical models, considering the conditions of injection time and needle position. Initially, the effect of injection time is studied on the 60° curved root canal. This study irrigates the root canal with normal saline flow under three different injection times 4, 8, and 12 s (Fig. 5a, b and 5c, respectively). shows the Streamlines of the irrigation flow for a 60° curved canal model in different injection times. Flow is entered into the root canal from the needle and washes the canal wall; afterward, it accumulates near the apex, with a velocity near zero at the range of stagnant dead water zone, and has a retrograde pattern without cleansing the lower part of the root canal. Finally, it comes out from the outlet plane at the top of the canal. In this manner, the faster injection causes achieving longer streamlines with higher flow velocity and more penetration depth of the flow.

**Fig. 6:**
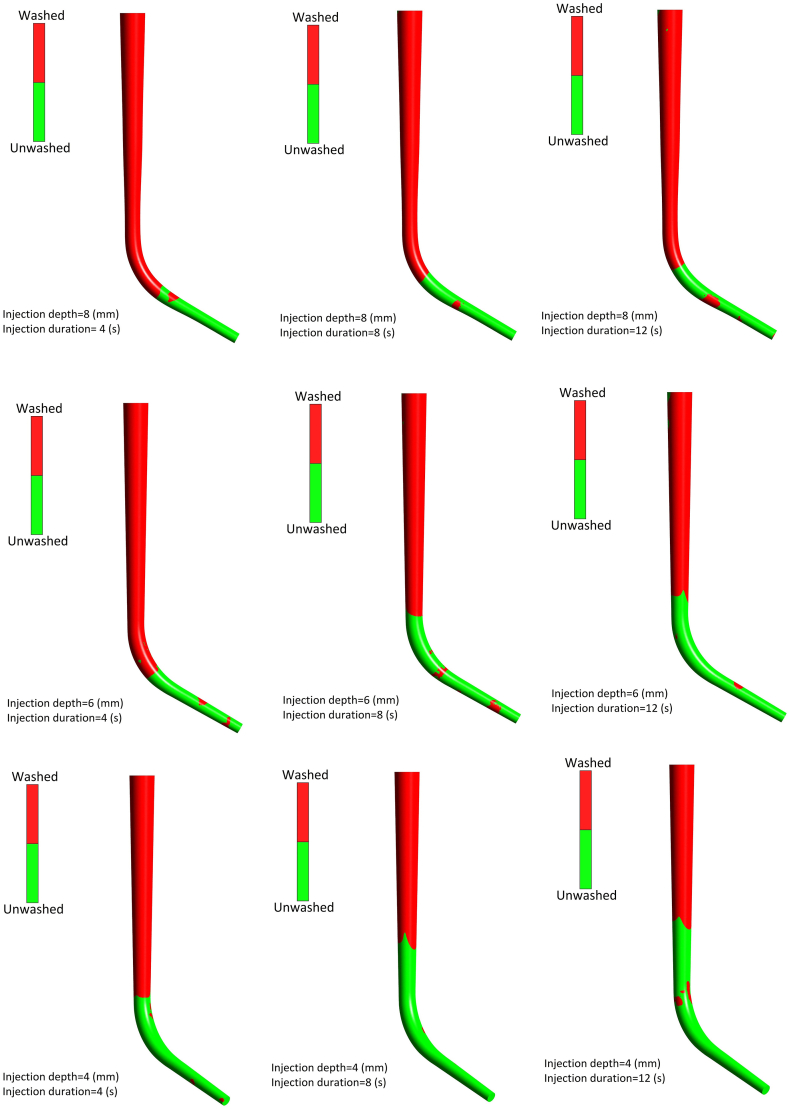
60° curved Root canal washed area under irrigation flow procedure with various injection positions and injection times.

**Figure (7):**
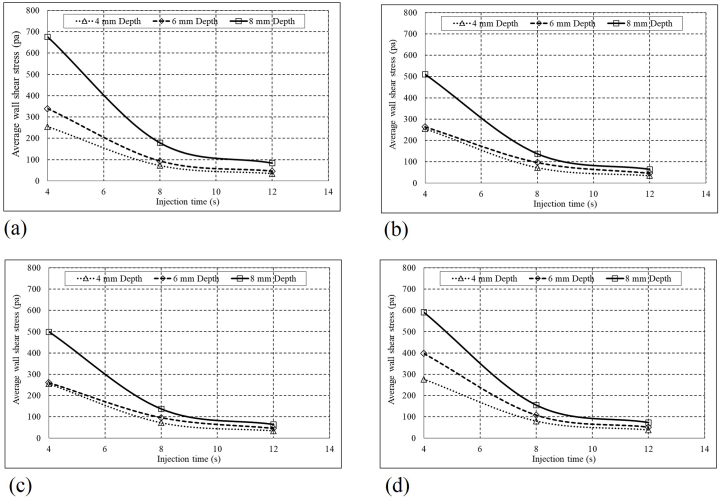
Root canal average wall shear stress under irrigation flow procedure with various injection positions and Injection times (a) 60°, (b) 30°, (c) 10° and (d) straight.

Following this, we aim to show that irrigation quality is significantly affected by changing the needle Position and injection velocity, which is directly associated with the injection time. A shorter injection time makes for higher injection velocity and more shear stress, increasing the flow power to remove debris particles. So, the irrigation quality can be investigated by evaluating the wall shear stress. This study assumes an area with wall shear stress higher than 1(Pa) as a washed area (Fig. 6a). shows the washed area of the 60° curved root canal to assess the irrigation quality in different positions of the needle and injection times. The average wall shear stress of the 60° curved root canal considering various needle positions and injection times are depicted in (Fig. 7a). Generally, the deeper position of the needle inlet increases the average wall shear stress. It can result that a deeper needle position with higher inlet velocity increases the average wall shear stress of the 60° root canal and causes more irrigation quality with a higher washed area.

Figs (7b, 7c, and 7d) show the average wall shear stress of the 30° and 10° curved root canals and the straight root canal, considering various needle positions and injection times. As it can be seen, in constant injection time and needle depth, a higher average shear wall occurs in straight root canals in comparison to 30° and 10° curved root canals. Comparison between Figs (6a, 6b, 6c, and 6d) shows that deeper needle position and faster injection results in higher average wall shear stress in both the straight root canal anatomy and 60° curved one case.

**Figure (8):**
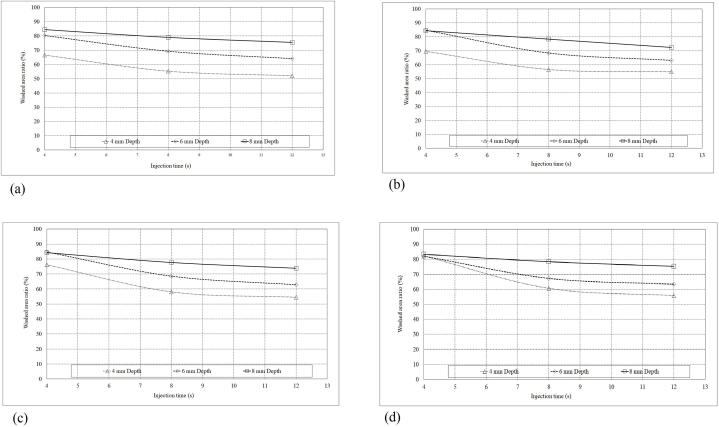
Root canal average washed area ratio under irrigation flow procedure with various injection positions and injection times (a) 60°, (b) 30°, (c) 10° and (d) straight.

The amount of washed area of the 60° curved root canal considering various needle positions and injection times are depicted in (Fig. 8). As seen in Fig 8a, decreasing the injection time from 12 (s) to 4 (s) through the cases of various needle depths as 8(mm), 6(mm), and 4 (mm), caused the 11.8 (%), 25.3 (%) and 27.6 (%) increase of the washed area respectively.

Figs (8b, 8c, and 8d) show the washed area ratio of the 30° and 10° curved root canals and the straight root canal, considering various needle positions and injection times. As can be seen, increasing the inlet velocity causes higher washed area in curved and straight root canals. This fashion is especially in cases with lesser root canal curves.

**Figure (9):**
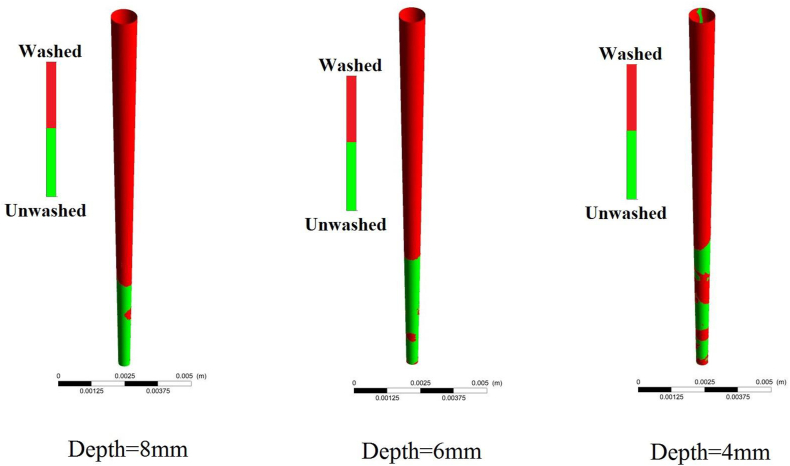
Straight root canal washed area under irrigation flow procedure with various injection position and injection time of 4(s).

According to (Fig. 8), in longer injection times (12 and 8 (s)), deeper needle position causes a higher washed area ratio. In the case of the injection time of 4 (s) and 23.54 (m s^−1^) as inlet velocity, the washed area ratio is significantly affected by the canal curve and needle position. The straight root canal under the injection time of 4(s) results in an 84 % washed area ratio considering various needle positions. Following this, washed area of a straight root canal with an injection time of 4(s) is illustrated (Fig. 9). This figure better implies the increase of irrigation quality by the deeper needle position.

**Figure (10):**
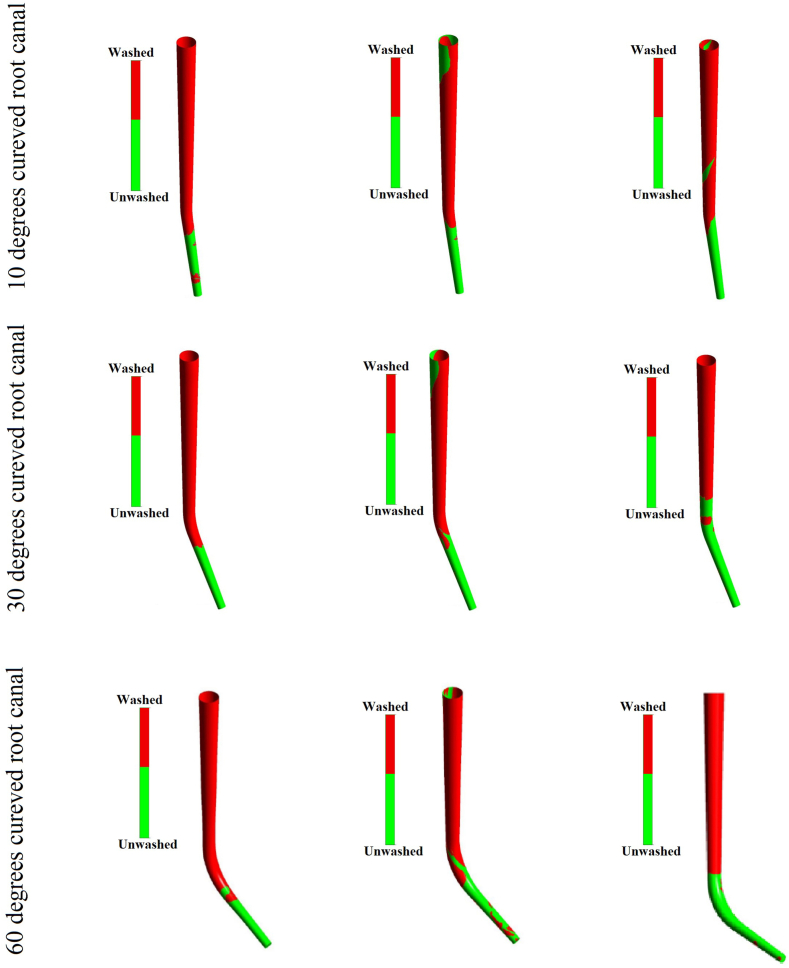
Curved root canal washed area under irrigation flow procedure with various injection position and injection time of 4(s).

The washed area of curved root canals considering various needle positions with a time of 4 (s) are depicted (Fig. 10). As well as increasing the root canal curve, faster injection, and deeper needle position are needed to achieve a higher washed area ratio.

In cases with an injection time of 4(s), the washed area ratio of curved root canals with a needle position of 8 and 6 (mm) is approximately the same. Whereas, at needle positions of 4 (mm), the washed area ratio of the straight, 10°, 30°, and 60° canals reached 84 %, 76 %, 70 %, and 68 %, respectively.

## 4 Discussion

Higher irrigation quality of root canal presages of better prognosis for root canal therapy [19,20]. Understanding the flowing nature of irrigant fluid allows a clinician to have a more reliable root canal therapy. Today, computational simulations have a major role in assessing the nature of physical phenomena. In dentistry, computational simulations are widely used by researchers and scientists [21]. These simulations are accessible, reliable, and inexpensive, especially in cases where in-vivo experiments are not allowed [22]. In endodontics, research is divided into two categories: root canals with idealized geometries and the patient-specific geometries of a natural tooth [23,24]. The study of idealized geometry allows researchers to understand canal nature better.

Conversely, the curvature of actual canals differentiates them from simplified geometries with straight conical root canals. The position of fluid injection and velocity, as well as curved canal geometry, can affect the flow regime in the root canal. It could be attributed to the formation of recirculating zones within the root canal. This research simulated the debridement process in curved and straight root canals with Normal saline injection using the 5 cc syringe (gauge 27). The irrigation quality of the curved root canals was studied considering 36 test cases with various positions of needle and injection time. Wall shear stress was considered the main parameter that determines the irrigation quality. Also, the washed area ratio of the root canals was calculated based on wall shear stress exceeding 1 Pa.

Results show that in the case of a straight root canal, more than 80% of the root canal's wall could be washed if the injection characteristics, like the injection time, be equal to 8 (s) with a needle depth of 8 (mm) or the injection time be 4 (s) even with the needle depth of 4 (mm). In cases of 10° and 30° curved root canals compared to a straight root canal, the same irrigation quality and washed area ratio could be obtained if the injection time is equal to 4 (s) with a needle depth of 6 (mm). In the case of the 60° curved root canal, the injection time of 4 (s) with a needle depth of 8 (mm) is essential. These findings agree with the study of Ghalandari et al. [13] which implies that wall shear stress increases with an increase in injection depth and solution concentration. Zhou et al. [25] investigated the effect of injection depth and canal curvature on the quality of irrigation. Similar to the present study, their results show that large curvature improves irrigation quality. Nevertheless, they conclude that an efficient short needle depth better affects irrigation. This may be attributed to a special turbulent flow regime in some needle depths to produce higher wall shear stress levels where the stagnation point may be in its efficient position.

In the other study, Liu et al. [26] investigated the effects of root canal curvature on the irrigant's flow pattern. They concluded that better efficacy was achieved by considering a safe-sided curved needle, deep positioning, and matching the needle's size and cross-section geometry of the canal. Hence, make a better adaptation of the needle with the canal walls. Of course, They used the curvature from zero to 30° in that study [26].

We analyzed the influence of canal curvature on irrigation quality. Results demonstrated that increasing the curvature from a straight root canal up to 10° and 30° canals decreases wall shear stress slightly. Interestingly, the average shear stress in 60° root canals increases dramatically. This phenomenon could be attributed to the formation of recirculation zones caused by increasing Reynold's number, leading to more flow turbulence and higher values of wall shear stresses [27]. This manner is not sensible for washed areas, where the straight root canal has higher values of the washed area.

Previous studies implied that changes in needle position in straight conical canals do not majorly impact the cleansing of the root canal. Nevertheless, the needle injection position can influence the wall shear stresses. This study found that changing the needle position affects the wall shear stresses, especially in deeper positions. This trend in straight and curved canals is similar.

In another approach, we investigated the influence of injection time. Injection time has a reverse relationship with initial injection velocity. The shorter the injection time, the higher the injection velocity. Results demonstrate that average wall shear stresses increase by decreasing the injection time. This increase is notable at the time of 4s and has a significant difference in 60 curved canals: the higher velocity and more tortuosity result in more significant turbulence and wall shear stress. The washed area has a similar behavior. It also increases by decreasing the injection time, but the differences are not as great as wall shear stresses. Interestingly, in a straight root canal at an injection time of 4s, the washed areas are almost identical for all three needle depths. It could be explained by symmetric streamlines at straight root canals that lesser affected by the needle depth at high velocities.

## 5 Conclusions

This research simulated a debridement procedure in curved and straight root canals with normal saline injection, considering the standard 5 cc syringe as a medical injector device. The effects of canal curvature, needle injection depth, and fluid injection velocity on the irrigation quality of root canals were investigated. The wall shear stresses greater than 1 Pa are selected as the irrigation criteria.

The following conclusions can be drawn:

i) Irrigation quality enhanced by increasing irrigation depth.
ii) Flow injection velocity increases the irrigation quality.
iii) Irrigation quality has a complex relationship with canal curvature. It depends on the formation of the recirculation zones in the root canal, which influence the wall shear stresses.

## Data availability statement

https://doi.org/10.17632/g3vzzdwbp9.1.

## CRediT authorship contribution statement

**Arash Izadi:** Writing – original draft, Supervision, Funding acquisition, Conceptualization. **Mohsen Lashkarbolok:** Writing – original draft, Software, Methodology, Formal analysis, Data curation. **Ezatolah Kazeminejad:** Writing – review & editing, Visualization, Formal analysis, Conceptualization. **Iman Tavakolinejad Kermani:** Writing – original draft, Validation, Formal analysis, Data curation. **Yaser Mesri:** Writing – review & editing, Validation, Project administration, Data curation.

## Declaration of competing interest

The authors declare that they have no known competing financial interests or personal relationships that could have appeared to influence the work reported in this paper.
