# Supplementary material for: Numerical investigation of injection characteristics on normal saline irrigation quality in curved root canals using computational fluid dynamics
Source: Heliyon. 2023 Dec 15;10(1):e23773. doi: 10.1016/j.heliyon.2023.e23773 (PMC10772184; doi:10.1016/j.heliyon.2023.e23773)
Supplement: Multimedia component 1 [file mmc1.docx]

**Appendix**

**Effects of Injection Characteristics on Normal Saline Irrigation Quality in Curved Root Canals Using Computational Fluid Dynamics**

Arash Izadi ^1^, Mohsen Lashkarbolok ^2^, Ezatolah Kazeminejad* ^3^, Iman Tavakolinejad Kermani ^4^, Yaser Mesri ^6^

^1 Dental Research Center, Golestan University of Medical Sciences, Gorgan, Iran^

^2 Department of Civil Engineering, Faculty of Engineering, Golestan University, Aliabad Katoul, Iran^

^3 Dental Research Center, Golestan University of Medical Sciences, Gorgan, Iran^

^Assistant Professor of Endodontics, Faculty of Dentistry, Golestan University of Medical Sciences, Gorgan, Iran^

^ORCID ID: 0000-0003-0557-767X^

^5 Department of Civil Engineering, Faculty of Engineering, Golestan University, Aliabad Katoul, Iran^

^6 Research Center for Biomedical Engineering, Ferdowsi University of Mashhad, Mashhad, Iran^

^7 Faculty of Dentistry, Golestan University of Medical Sciences, Gorgan, Iran^

**^*Corresponding author^**^, Ezatolah Kazeminejad; E-mail:^ [^Dr.kazeminejad@goums.ac.ir^](mailto:Dr.kazeminejad@goums.ac.ir)

Table 1. Curvature categories and injection characteristics of the studied cases

| **Case** | **degree of the root canal curvature** | **Needle location**  **(mm)** | **Inlet velocity**  **(m s^-1^)** | **injection time**  **(m s^-1^)** |
| --- | --- | --- | --- | --- |
| 1 | 0 | 4 | 23.54 | 4 |
| 2 | 0 | 4 | 11.77 | 8 |
| 3 | 0 | 4 | 7.84 | 12 |
| 4 | 0 | 6 | 23.54 | 4 |
| 5 | 0 | 6 | 11.77 | 8 |
| 6 | 0 | 6 | 7.84 | 12 |
| 7 | 0 | 8 | 23.54 | 4 |
| 8 | 0 | 8 | 11.77 | 8 |
| 9 | 0 | 8 | 7.84 | 12 |
| 10 | 10 | 4 | 23.54 | 4 |
| 11 | 10 | 4 | 11.77 | 8 |
| 12 | 10 | 4 | 7.84 | 12 |
| 13 | 10 | 6 | 23.54 | 4 |
| 14 | 10 | 6 | 11.77 | 8 |
| 15 | 10 | 6 | 7.84 | 12 |
| 16 | 10 | 8 | 23.54 | 4 |
| 17 | 10 | 8 | 11.77 | 8 |
| 18 | 10 | 8 | 7.84 | 12 |
| 19 | 30 | 4 | 23.54 | 4 |
| 20 | 30 | 4 | 11.77 | 8 |
| 21 | 30 | 4 | 7.84 | 12 |
| 22 | 30 | 6 | 23.54 | 4 |
| 23 | 30 | 6 | 11.77 | 8 |
| 24 | 30 | 6 | 7.84 | 12 |
| 25 | 30 | 8 | 23.54 | 4 |
| 26 | 30 | 8 | 11.77 | 8 |
| 27 | 30 | 8 | 7.84 | 12 |
| 28 | 60 | 4 | 23.54 | 4 |
| 29 | 60 | 4 | 11.77 | 8 |
| 30 | 60 | 4 | 7.84 | 12 |
| 31 | 60 | 6 | 23.54 | 4 |
| 32 | 60 | 6 | 11.77 | 8 |
| 33 | 60 | 6 | 7.84 | 12 |
| 34 | 60 | 8 | 23.54 | 4 |
| 35 | 60 | 8 | 11.77 | 8 |
| 36 | 60 | 8 | 7.84 | 12 |
